# A pathomic approach for tumor-infiltrating lymphocytes classification on breast cancer digital pathology images

**DOI:** 10.1016/j.heliyon.2023.e14371

**Published:** 2023-03-09

**Authors:** Mario Verdicchio, Valentina Brancato, Carlo Cavaliere, Francesco Isgrò, Marco Salvatore, Marco Aiello

**Affiliations:** a IRCCS SYNLAB SDN, Via E. Gianturco 113, Naples, 80143, Italy; b Department of Electrical Engineering and Information Technologies, University of Naples Federico II, Claudio 21, Naples, 80125, Italy

**Keywords:** Digital pathology, Pathomics, Tumor infiltrating lymphocytes, Breast cancer, Machine learning

## Abstract

**Background and objectives:**

The detection of tumor-infiltrating lymphocytes (TILs) could aid in the development of objective measures of the infiltration grade and can support decision-making in breast cancer (BC). However, manual quantification of TILs in BC histopathological whole slide images (WSI) is currently based on a visual assessment, thus resulting not standardized, not reproducible, and time-consuming for pathologists. In this work, a novel pathomic approach, aimed to apply high-throughput image feature extraction techniques to analyze the microscopic patterns in WSI, is proposed. In fact, pathomic features provide additional information concerning the underlying biological processes compared to the WSI visual interpretation, thus providing more easily interpretable and explainable results than the most frequently investigated Deep Learning based methods in the literature.

**Methods:**

A dataset containing 1037 regions of interest with tissue compartments and TILs annotated on 195 TNBC and HER2+ BC hematoxylin and eosin (H&E)-stained WSI was used. After segmenting nuclei within tumor-associated stroma using a watershed-based approach, 71 pathomic features were extracted from each nucleus and reduced using a Spearman's correlation filter followed by a nonparametric Wilcoxon rank-sum test and least absolute shrinkage and selection operator. The relevant features were used to classify each candidate nucleus as either TILs or non-TILs using 5 multivariable machine learning classification models trained using 5-fold cross-validation (1) without resampling, (2) with the synthetic minority over-sampling technique and (3) with downsampling. The prediction performance of the models was assessed using ROC curves.

**Results:**

21 features were selected, with most of them related to the well-known TILs properties of having regular shape, clearer margins, high peak intensity, more homogeneous enhancement and different textural pattern than other cells. The best performance was obtained by Random-Forest with ROC AUC of 0.86, regardless of resampling technique.

**Conclusions:**

The presented approach holds promise for the classification of TILs in BC H&E-stained WSI and could provide support to pathologists for a reliable, rapid and interpretable clinical assessment of TILs in BC.

## 1 Introduction

**Fig. 1:**
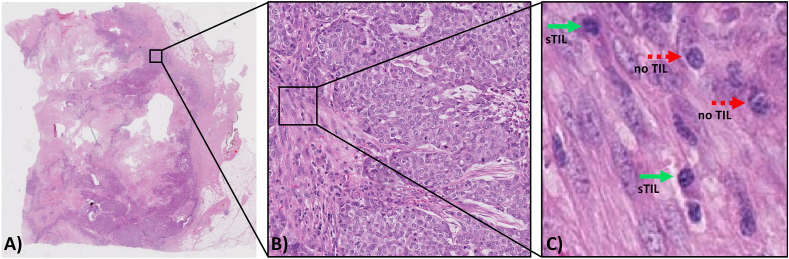
Identifying stromal Tumor Infiltrating Lymphocyte (TIL) regions in a H&E-stained WSI of breast cancer. (A) H&E-stained WSI of breast cancer. (B) Example of a region of tissue. (C) Example of stromal TILs (green solid arrow) and cells that are no TILs (red dotted arrow). The image shows how it can be challenging to visually differentiate TILs from other cells. (For interpretation of the references to color in this figure legend, the reader is referred to the Web version of this article.)

The treatment of breast cancer (BC) is largely determined by the biology of the tumor. It is becoming more evident that the patient's immunity can be an important indicator of what treatment is needed and how their own immune system can significantly contribute to their chances of long-term survival [1]. In recent years, the role of the tumor microenvironment (TME) has received increasing attention in the immuno-oncology scientific community, focusing on the interaction between tumor cells and the host immune system [2]. Tumor-infiltrating lymphocytes (TILs), namely lymphocytes and plasma cells that have invaded the tumor tissue, are proving to be an important biomarker in cancer patients as they can play a role in killing tumor cells, particularly in some types of BC. In recent years, several studies have shown the predictive and prognostic value of visually scored TILs in triple-negative BC (TNBC) and human epidermal growth factor receptor 2 (HER2+) BC, making TILs a powerful clinical biomarker [3,4]. Therefore, accurate identification and measurement of TILs can support less aggressive target treatments, particularly immunotherapy, reducing the need for more invasive options like chemotherapy. Currently, the degree of TILs infiltration is assessed by simple visual evaluation of hematoxylin and eosin (H&E)-stained whole slide images (WSI) of tumor sections, following recommendations from the International TILs Working Group (TILs-WG) [5,6]. According to TILs-WG guidelines, the boundaries of the invasive tumor must be identified, and only TILs inside those boundaries were evaluated. In particular, the TILs-WG suggests only considering stromal TILs (sTILs), which are defined as mononuclear hosts immune cells (predominantly lymphocytes) present within the tumor margin and located within the stroma among carcinoma cells without directly contacting or infiltrating tumor cell nests (Fig. 1(A-C)). This is because sTILs are more prevalent, more variable in amount and have been demonstrated to be more reproducibly assessed when compared to intratumoral TILS (iTILs), defined as lymphocytes within nests of carcinoma characterized by cell-to-cell contact with no presence of stroma among them [7]. sTILs are then reported as a percentage, which refers to the percentage of stromal area occupied by mononuclear inflammatory cells over the total within-tumor stromal area [7]. Specifically, the TIL score is manually estimated as the percentage of total tumor-associated stromal area occupied by TILs, where areas of necrosis, ductal and lobular carcinoma in situ (DCIS/LCIS), and normal breast tissue are excluded [5]. However, despite these successful standardization efforts and the rising amounts of evidence concerning the association of TILs with favorable prognosis [8], manual quantification of TILs has several drawbacks: is time-consuming, limited in precision, requires extensive training by pathologists, inter-observer variability will likely remain an issue given the difficulty in quantitatively evaluating histological properties, and lacks the ability to evaluate more complex properties such as TIL distribution patterns or features associated with tissue phenotype. Therefore, this lack of standardized, objective, and effective TILs quantification strategies make TILs marker still not considered robust enough for being used in clinical practice [5]. In several TIL studies, pathologists count by eye the quantity of lymphocytes within a limited area, an obviously very error-prone and time-consuming task [3]. To favor the reporting of TILs in BC it is therefore necessary to provide the pathologists with automated image analysis tools. The availability of a computerized image analysis scheme for automated quantification of TILs will enable the development of an inexpensive image-based system for predicting disease survival and outcome. In particular, computational image analysis approaches are crucial for improving TILs quantification accuracy, saving time, enabling the analysis of more complex spatial patterns, and providing standardized metrics for validation by expert pathologists and regulatory agencies [[9], [10], [11]].

Over the last decade, the rapidly increasing technical advancements in the field of digital pathology enabled the development of increasingly advanced computerized image analysis approaches for automating manual tasks such as TILs quantification and addressing their associated limitations. Different automated TILs quantification approaches have been proposed, ranging from those based on classical image processing methods, where cell boundaries are identified and the resulting objects classified according to certain properties, to deep learning (DL)-based approaches that directly classify cells without the need for explicit segmentation [9,[12], [13], [14]]. Although the most common strategies proposed to quantify TILs are those exploiting DL supervised techniques, achieving high quality results with this group of methods requires large scale dataset for training with precisely annotated masks accurately representing the cell borders (in case of semantic segmentation approaches such as the U-Net [15]) or the exact locations (in case of object-detection approaches such as Fast *R*–CNN [16] and YOLO [17]) of TILs in training images. Moreover, DL-based approaches are mostly neither interpretable nor easily explainable [18,19], and fail to aid pathologists with novel insights on the tissue since they lack in providing a structured representation of the input image data.

The emerging and rapidly expanding field of pathomics aims to apply high-throughput image feature extraction techniques to interrogate the microscopic patterns in pathologic data, especially from H&E-stained sections [20]. The founding hypothesis in support of the use of pathomics in medical care is that quantitative features derived from digital pathology images could give additional information in relation to the underlying biological processes compared to the visual interpretation of the image as a picture, which is the traditional way of interpreting images. In this context, features extracted from pathomic analysis could be a promising tool for automatically identifying TILs in BC H&E images since they may contain information helping to discover novel biological associations that could be useful for TILs detection [20]. Moreover, they provide more easily interpretable and explainable results than those obtained using DL-based approaches for TILs detection [18,21,22]. Only a few previous studies aimed at detecting TILs using quantitative features from H&E images of different cancer types [12,23,24], while most studies aimed at developing DL-based computational TIL assessment methods broadly following some or all steps of visual TILs-WG guidelines [21,22,25,26]. However, automated TILs quantification methods are still far from reaching clinical practice for many reasons (e.g., requirement of more rigorous design, greater sample size, more standardized survival analyses, high demand for powerful infrastructures for training data etc.) and studies contributing to achieving a quantitative, automatic, interpretable, and as simple as possible (both considering the implementation and the hardware requirements) are needed.

In light of this, this study aims to develop an approach based on a comprehensive set of hand-crafted pathomic features to automatically classify TILs in H&E-stained WSI of BC sections. It is worth remarking that pathomic features are explicit, therefore easy to interpret, as opposed to the DL approach. Several predictive models built using these features will be evaluated to study how different classifiers influence the results.

## 2 Materials and methods

**Fig. 2:**
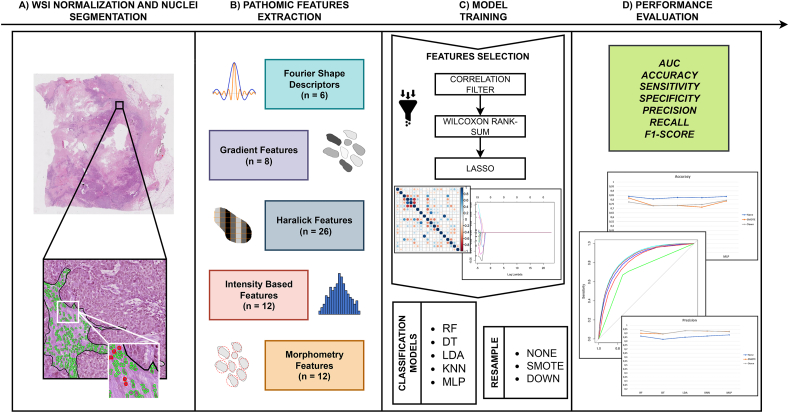
Workflow of the approach developed in the study. Starting from H&E images from HER2+ and TN breast cancer, the approach starts with the segmentation of nuclei within tumor-associated associated stroma (A). Pathomic features were then extracted from cell segmentation within tumor-associated stroma (B). After the feature selection step, five classification models were trained (C) and their performance evaluated (D).

In this section, data used in this study as well as the preprocessing methods and the algorithm for nuclei segmentation are introduced. Moreover, the extraction of pathomic features, feature selection and classifier modeling are described in detail. Finally, the metrics used for the evaluation are provided. The overall methodological workflow of this study is shown in Fig. 2(A-D).

### 2.1 Dataset

A public dataset of diagnosed TNBC and HER2+ BC patients was used https://tiger.grand-challenge.org/. In particular, for the development of the pipeline the ‘WSIROIS’ dataset was employed. This dataset consists of 195 WSIs of core-needle biopsies and surgical resections of BC patients with annotated tissue compartments and TILs, combining cases from three sources.

• TCGA: TNBC cases from TCGA-BRCA [27] archive (n = 151). The annotations provided for this dataset were generated by adapting the publicly available BCSS (https://bcsegmentation.grand-challenge.org/) and NuCLS (https://nucls.grand-challenge.org/ [24])datasets.
• RUMC: 26 cases of TNBC and HER2+ cases from Radboud University Medical Center (Netherlands), with annotations by a panel of board-certified breast pathologists.
• JB: 18 cases of TNBC and HER2+ cases from Jules Bordet Institute (Belgium). Annotations for these were made by a panel of board-certified breast pathologists.

All data were provided at 20 × magnification (resolution of 0.5 micron-per-pixel) for processing. All images have ROI-level annotation masks for use in processing. Concerning annotations corresponding to tissue compartments, each image had ROI-level annotations of the following tissue classes: invasive tumor, tumor-associated stroma, in-situ tumor, healthy glands, necrosis not in-situ, inflamed stroma and other. ROIs of tissue compartments were manually annotated using polygons. TILs were manually annotated using point annotations and then squared bounding boxes of 8 × 8 μm were constructed centered on the point annotation [28]. Five breast pathologists independently performed tissue and TILs annotations and any disagreement was solved by consensus. The classes ‘tumor-associated stroma’ and ‘inflamed stroma’ were merged to form a ‘stroma’ class. Then, all other classes were set to zero. In this way, masks only consisted of background and stroma, and TILs. ROIs without stromal tissue annotation were excluded.

### 2.2 Preprocessing

Stain normalization was performed in the preprocessing step to each image using the method proposed by Vahadane et al. [29]. For each image, this method involves a stain separation by sparse non-negative matrix factorization, in which the image is decomposed into sparse and non-negative stain density maps, followed by a structure-preserving color normalization step aimed at altering only the image color appearances while preserving the structure [29]. Nuclear segmentation was performed on H&E-stained slides to recognize objects through a watershed-based cell detection method. Of note, nuclear segmentation is a crucial but challenging task in pathology image analysis, and the search for a robust, practically useable nucleus segmentation module is still ongoing [[30], [31], [32]]. Various different approaches have been proposed, ranging from relatively simple thresholding techniques (e.g. Otsu [33], watershed [34]) to more advanced methods, such as, for instance, those based on deep learning [30]. In this study, a watershed-based method was used for its documented advantages, namely simplicity, speed and the ease with which parameters can be adjusted and fine-tuned. This method applies a set of mathematical operations (fast radial symmetry transform and regional minima) at different scales to identify candidate locations for nuclei [34] and is part of the most widely used nuclear segmentation methods among pathologists due to its implementation within the most popular digital pathology tools (e.g. Cell Profiler [35], Qupath [26], ImageJ [36], OpenCV [37]).

The cell detection function from the analysis module of QuPath was used to perform watershed-based nuclei segmentation within tumor-associated stroma [34,[38], [39], [40]]. The setup parameter was set as hematoxylin OD for the detection image, with a pixel size of 0.5 μm. Nucleus parameters were set as follows: background radius: 8 μm; median filter radius: 0 μm; σ: 1.5 μm; minimum cell area: 10 μm^2^; maximum cell area: 400 μm^2^ [41]. For intensity parameters, the threshold was set to 0.1, and the maximum background intensity was set to 2. An expert microscopist verified the accuracy of the automatic cell detection.

### 2.3 Feature extraction and selection

71 pathomic features were extracted using the open-source HistomicsTK package https://github.com/DigitalSlideArchive/HistomicsTK/from each segmented nucleus. The extracted radiomics features were categorized into five groups: 6 Fourier Shape Descriptors (FSD) features, 8 gradient features, 26 Haralick features, 12 intensity-based features, 19 morphometry features. The computing algorithms can be found at https://digitalslidearchive.github.io/HistomicsTK/histomicstk.features.html. Features selection was performed using a correlation filter based on the absolute values of pairwise Spearman's correlation (ρ) coefficient to reduce feature redundancy and prevent model overfitting (To reduce the dimension of features and solve the overfitting problem). The threshold for ρ was set to 0.9. Briefly, if two features had ρ > 0.9, the function looks at the mean absolute correlation of each variable, and the variable with the largest mean absolute correlation is removed. A further feature reduction through a univariate analysis using a nonparametric Wilcoxon rank-sum test was performed to investigate their statistical significance with respect to the outcome (TIL/noTIL). The significantly different features (p < 0.05) were then selected. The optimal feature set from the remaining features was selected using the least absolute shrinkage and selection operator (LASSO) algorithm method, which was used to identify the most outcome-related features. The LASSO is a regularization technique used to minimize the number of non-zero elements and make the solution unique. In the LASSO algorithm, the shrinkage parameter lambda was identified when the misclassification error was smallest in 5-fold cross-validation [42,43]. The surviving features were used to build pathomic-based prediction models. Feature selection procedures were implemented in R studio software, version 4.0.2 (downloadable from http://www.R-project.org).

### 2.4 Data processing

To avoid the predominance of features with the largest scale in the analysis, z normalization was performed on the training set prior model training and applied to the test set. According to z normalization, each feature was normalized as $\frac{\left(x-x^{\prime }\right)}{s}$ where x, x’, and s are the feature, the mean, and the standard deviation, respectively.

### 2.5 Model building and performance evaluation

Before data processing, data were randomly split into training (70%) and testing sets (30%) but stratified to ensure that the ratio of positive samples to negative samples was the same between the training and test set. Five different models were used to evaluate the discrimination power of pathomic features: Linear Discriminant Analysis (LDA), K-Nearest Neighbors (KNN), Decision Tree (DT), Random Forest (RF), Multi-layer Perceptron (MLP). Five common classifiers from different families were chosen to see which of them provides the best classification performance. K-Fold cross-validation (CV) with K = 5 was applied to the training data set for model selection (hyperparameter tuning). Concerning model parameters, the number of neighbors k in k-NN was selected from Refs. [5,7,9]; the number of trees to grow and the number of variables randomly sampled as candidates at each split was set to *ntree* = 500 and *mtry* = $\sqrt{number\;of\;predictors}$, respectively; the confidence factor for DT was chosen from cp ∈ [0.1, 0.5]; the number of hidden layers in MLP was selected from hl ∈ [1,5] no tuning parameters were needed for LDA. Additionally, to overcome the imbalance in the distribution of the TILs condition, models were also trained using both downsampling and a synthetic minority oversampling technique (SMOTE) procedure on the training dataset to achieve balanced classes. The downsampling technique is a resampling method that decreases the size of the majority class to be the same or closer to the minority class size by just taking out a random sample. On the other hand, SMOTE is a hybrid resampling method that downsamples the majority class, and artificially generates new examples of the minority class using the nearest neighbors of these cases. Furthermore, the majority class examples are also under-sampled, leading to a more balanced dataset [44]. Receiver operating characteristic (ROC) curve analysis was applied to evaluate the predictive performance of the classification models (in all three settings: not resampled, downsampled, and SMOTE-sampled training set), and the area under the curve (AUC) was calculated. Accuracy (Eq. (1)), sensitivity or recall (Eq. (2)), specificity (Eq. (3)), precision (Eq. (4)), and F1-score (Eq. (5)) were employed to evaluate the diagnostic performance of resulting models in the hold-out test set. The associated formulas are listed below:

(1) $$Accuracy=\frac{TP+TN}{TP+TN+FP+FN}$$

(2) $$Sensitivity=\frac{TP}{TP+FN}$$

(3) $$Specificity=\frac{TN}{TN+FP}$$

(4) $$Precision=\frac{TP}{TP+FP}$$

(5) $$F1=\frac{precision\;*\;recall}{precision+recall}$$

where TP = True Positive, TN = True Negative, FP = False Positive, FN = False Negative.

The AUC-ROCs were statistically compared between different classifiers and between different resampled strategies using DeLong test with Bonferroni correction. P values less than 0.05 were considered significant. Models building and evaluation were implemented using Caret package in R studio software, version 4.0.2, downloadable from http://www.R-project.org.

## 3 Results

A total of 1037 ROIs with tissues compartments and TILs annotated were considered. Nuclei segmentation resulted in 92,141 nuclei, of which 20,111 TILs.

**Fig. 3:**
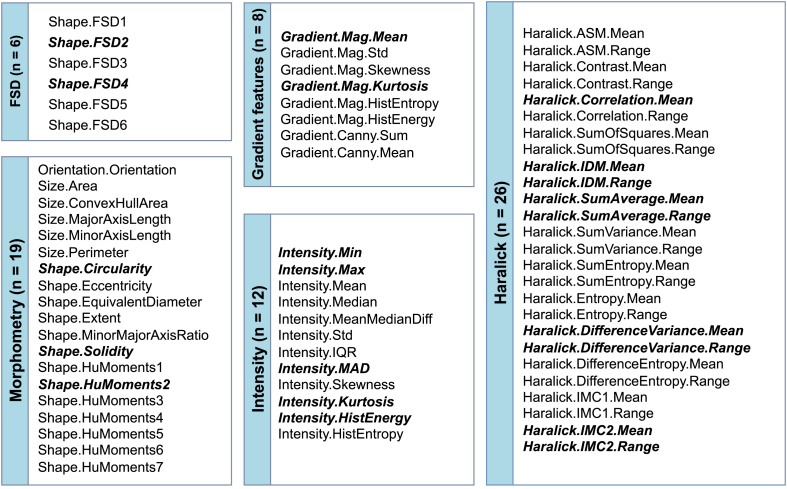
Selected pathomic features (n = 21) after the three steps of feature selection. Surviving features were reported in bold. Abbreviations: FSD = Fourier Shape Descriptor; MAD = Median Absolute Deviation; IDM = Inverse Difference Moment; IMC = Information Measure of Correlation.

Selection of pathomic features returned 21 features, of which 2 from FSD group, 2 from gradient group, 9 from Haralick group, 3 from morphometry group and 5 from intensity group (Fig. 3).

**Fig. 4:**
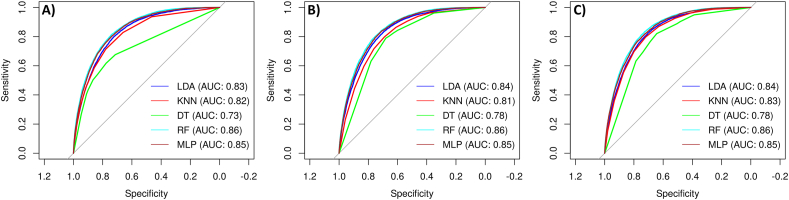
Receiver Operating Characteristic (ROC) curves of prediction models without resampling (A), with SMOTE-resampling (B) and with downsampling (C). Abbreviations: LDA = Linear Discriminant Analysis; KNN = K-Nearest Neighbour; DT = Decision Tree; RF = Random Forest; MLP = Multi-layer Perceptron.

**Fig. 5:**
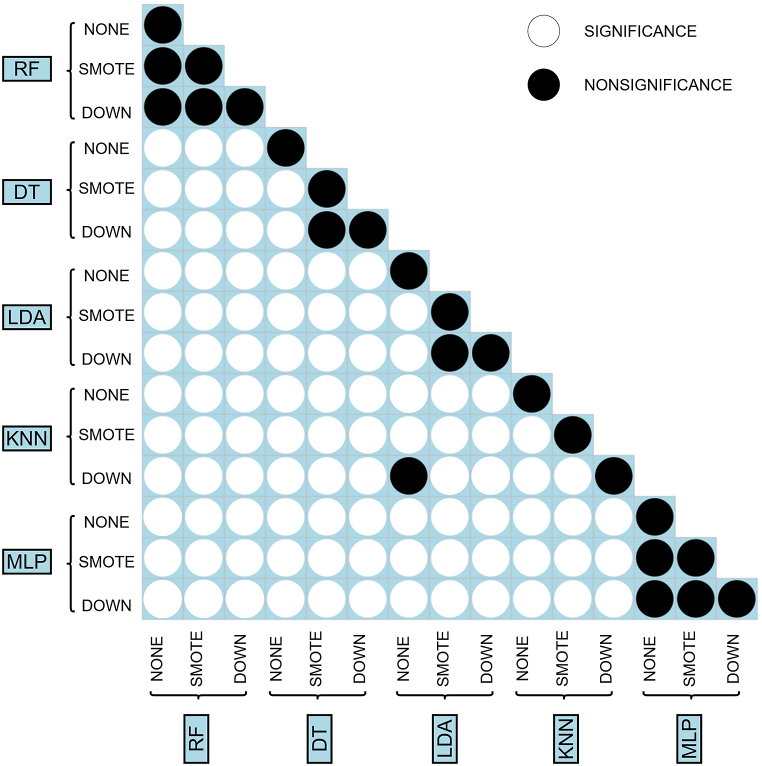
Comparison of AUCROCs between different classification models and different resampling techniques. Abbreviations: SMOTE = synthetic minority oversampling technique; LDA = Linear Discriminant Analysis; KNN = K-Nearest Neighbour; DT = Decision Tree; RF = Random Forest; MLP = Multi-layer Perceptron.

Table 1, Table 2 reported the predictions of the five built machine learning models. Moreover, Fig. 4 shows the ROC curves representing performances of each classifier on the test set, both in the original setting, in the downsampled setting and the SMOTE-sampled setting. The performance of RF were the best according to AUC and the DeLong test, with or without applying resampling techniques, both in the training (*AUCROC*_*RF*_ = 0*.*857–0.858) and the test set (*AUCROC*_*RF*_ = 0*.*855–0.857). DeLong test revealed that all except 9 AUC comparisons were significant. It can be observed that the use of resample technique did not significantly affect MLP and RF prediction performances. In addition, performances of KNN with downsampling were not significantly different from those of KNN with smote resampling. The same behavior was observed for LDA. In addition, classification performances of KNN with downsampling were not significantly different from those of LDA without resampling. Refer to Fig. 5 for an overview of DeLong test results. Of note, although the sensitivity of models with SMOTE or down resampling in training and validation sets declined, the specificity improved greatly. However, RF produced the best classification performances, suggesting that these models could potentially be employed to discriminate TILs from noTILs. Comparing the AUCROC for SMOTE, downsampling, and the original data, applying resampling does not improve performances (Refer to Table 2).

**Table 1:** Average prediction performance of different pathomic models for classifying TILs from noTILs in the training set with 5-fold CV. Bold indicates best performing models. Abbreviations: AUCROC = Area Under the Receiver Operating Characteristic curve; SMOTE = synthetic minority oversampling technique; LDA = Linear Discriminant Analysis; KNN = K-Nearest Neighbour; DT = Decision Tree; RF = Random Forest; MLP = Multi-layer Perceptron.

Models	Resample	AUCROC	Accuracy	Sensitivity (Recall)	Specificity	Precision	F1-score
**RF**	**None**	**0.8576 ± 0.0029**	**0.8306 ± 0.0024**	**0.9398 ± 0.0025**	**0.4386 ± 0.0036**	**0.8572 ± 0.0009**	**0.8966 ± 0.0015**
	**SMOTE**	**0.8541 ± 0.0027**	**0.8162 ± 0.0034**	**0.8744 ± 0.0033**	**0.6077 ± 0.0083**	**0.8888 ± 0.0022**	**0.8815 ± 0.0023**
	**Down**	**0.8572 ± 0.003**	**0.7617 ± 0.0025**	**0.7486 ± 0.0029**	**0.8086 ± 0.0073**	**0.9335 ± 0.0023**	**0.8309 ± 0.0019**
DT	None	0.7383 ± 0.0046	0.8085 ± 0.0018	0.9413 ± 0.0133	0.3322 ± 0.0556	0.8351 ± 0.0096	0.8849 ± 0.0006
	SMOTE	0.7712 ± 0.0165	0.7162 ± 0.01	0.7013 ± 0.0216	0.7699 ± 0.032	0.9164 ± 0.008	0.7943 ± 0.0106
	Down	0.7702 ± 0.021	0.7012 ± 0.0113	0.6763 ± 0.0203	0.7905 ± 0.027	0.9207 ± 0.0075	0.7796 ± 0.0115
LDA	None	0.8365 ± 0.0019	0.813 ± 0.0016	0.9467 ± 0.003	0.3332 ± 0.0069	0.8359 ± 0.0011	0.8878 ± 0.0011
	SMOTE	0.8395 ± 0.0024	0.725 ± 0.003	0.6949 ± 0.005	0.833 ± 0.0069	0.9372 ± 0.0021	0.798 ± 0.0029
	Down	0.8392 ± 0.0024	0.7237 ± 0.0026	0.693 ± 0.0037	0.8335 ± 0.0065	0.9372 ± 0.0022	0.7968 ± 0.0023
KNN	None	0.8232 ± 0.0036	0.817 ± 0.0022	0.9176 ± 0.002	0.4563 ± 0.0067	0.8582 ± 0.0015	0.8869 ± 0.0014
	SMOTE	0.8151 ± 0.0025	0.7159 ± 0.0039	0.6905 ± 0.0048	0.8069 ± 0.0079	0.9277 ± 0.0027	0.7917 ± 0.0033
	Down	0.8335 ± 0.0032	0.7385 ± 0.0056	0.7208 ± 0.0061	0.8017 ± 0.0044	0.9288 ± 0.002	0.8117 ± 0.0046
MLP	None	0.8524 ± 0.0035	0.8253 ± 0.0034	0.9279 ± 0.0093	0.4569 ± 0.0313	0.8598 ± 0.0059	0.8925 ± 0.0024
	SMOTE	0.8518 ± 0.0024	0.7628 ± 0.0254	0.7568 ± 0.0461	0.7844 ± 0.0492	0.9271 ± 0.0117	0.8324 ± 0.0235
	Down	0.8534 ± 0.0029	0.7559 ± 0.0196	0.743 ± 0.0344	0.8023 ± 0.0341	0.9313 ± 0.0085	0.826 ± 0.0186

**Table 2:** Average prediction performance of different pathomic models for classifying TILs from noTILs in the test set. Bold indicates best performing models. Abbreviations: AUCROC = Area Under the Receiver Operating Characteristic curve; CI = Confidence Interval; SMOTE = synthetic minority oversampling technique; LDA = Linear Discriminant Analysis; KNN = K-Nearest Neighbour; DT = Decision Tree; RF = Random Forest; MLP = Multi-layer Perceptron.

Models	Classifier	AUCROC (95% CI)	Accuracy (95% CI)	Sensitivity (Recall)	Specificity	Precision	F1-score
**RF**	**None**	**0.8567 (0.8518, 0.8567)**	**0.8296 (0.8251, 0.834)**	**0.9395**	**0.4373**	**0.8563**	**0.8960**
	**SMOTE**	**0.8552 (0.8503, 0.8552)**	**0.8158 (0.8112, 0.8204)**	**0.8733**	**0.6107**	**0.8890**	**0.8811**
	**Down**	**0.8569 (0.8521, 0.8569)**	**0.7617 (0.7567, 0.7667)**	**0.7475**	**0.8125**	**0.9344**	**0.8305**
DT	None	0.8182 (0.8124, 0.8182)	0.8003 (0.7955, 0.805)	0.9107	0.4062	0.8455	0.8769
	SMOTE	0.7751 (0.7693, 0.7751)	0.7037 (0.6983, 0.7091)	0.6796	0.7896	0.9202	0.7818
	Down	0.8284 (0.823, 0.8284)	0.6825 (0.677, 0.688)	0.6438	0.8206	0.9276	0.7601
LDA	None	0.834 (0.8286, 0.834)	0.8134 (0.8087, 0.818)	0.9467	0.3376	0.8361	0.8880
	SMOTE	0.8372 (0.8319, 0.8372)	0.7225 (0.7172, 0.7278)	0.6906	0.8365	0.9378	0.7954
	Down	0.8367 (0.8314, 0.8367)	0.722 (0.7166, 0.7272)	0.6903	0.8352	0.9373	0.7950
KNN	None	0.8182 (0.8124, 0.8182)	0.8136 (0.809, 0.8182)	0.9159	0.4487	0.8557	0.8848
	SMOTE	0.8097 (0.8039, 0.8097)	0.7125 (0.7072, 0.7179)	0.6883	0.7991	0.9244	0.7891
	Down	0.8284 (0.823, 0.8284)	0.7362 (0.7309, 0.7413)	0.7189	0.7977	0.9269	0.8098
MLP	None	0.8491 (0.8439, 0.8491)	0.823 (0.8185, 0.8275)	0.9476	0.3782	0.8447	0.8932
	SMOTE	0.8481 (0.8431, 0.8481)	0.7662 (0.7612, 0.7712)	0.7632	0.7768	0.9243	0.7632
	Down	0.8503 (0.8453, 0.8503)	0.7476 (0.7424, 0.7527)	0.7290	0.8140	0.9333	0.8186

## 4 Discussion

Growing evidence has revealed that TILs are an important prognostic positive biomarker in BC patients [3]. Despite standardization efforts, the quantification of TILs is still based on a visual assessment, thus being time-consuming, labor-intensive, requiring large experience and expertise, prone to the inter-pathologist discrepancy, and associated with a mere qualitative evaluation of histological properties [5]. Therefore, the TILs marker is still not considered robust enough to be used in clinical practice, mainly because there are no standardized, objective, and effective TILs quantification strategies. Despite efforts from many researchers, the pathologist does not regularly assess the TIL infiltration due to a lack of automatic image analysis tools. Automatic detection of TILs using computational image analysis approaches could contribute to developing objective and precise mechanisms to measure the infiltration grade of BC, with a look towards the possibility of reaching a standardized TILs assessment that pathologists could then use in clinical practice for decision-making and treatment planning. Most current literature on automated TILs quantification relies on DL-based methods, which could be highly effective for the task, but due to their not immediate interpretability, fail to aid pathologists with novel insights on the tissue. Given the need for studies contributing to achieving a quantitative, automatic, interpretable, and as simple as possible TILs evaluation, a pathomic-based approach for classifying TILs on BC digital pathology images was proposed, exhibiting several types of interpretable features for automatic TILs classification. Results showed that some specific pathomic features extracted from nuclei in tumor-associated stromal regions of H&E images of BC could classify if nuclei were TILs or not. In particular, several machine learning models with selected pathomic features that could classify sTILs with high prediction performances after a feature selection process were developed. Considering the prediction performances of ML models, RF revealed the highest predictive value among different classifiers, with AUCs reaching 86%. Downsampling and SMOTE sub-sampling techniques were applied to tackle the unbalanced data problem and showed improvement in specificity. Machine learning algorithms tend to sacrifice the minority group for an unbalanced dataset to achieve higher accuracy [45]. Although both two subsampling methods improved specificity, SMOTE was able to enhance AUC in a balanced way: while maintaining high sensitivity, it was also able to increase specificity significantly. In addition, SMOTE has been shown to be robust to the variation of unbalanced ratios with various classifiers [44]. Previous studies aimed at detecting TILs using visual features from H&E images of different cancer types [23,24,46]. Basavanhally et al. [23] developed an approach combining a region-growing algorithm and Markov random fields to identify lymphocytes in HER2+ BC images. Kuo et al. [47], in a study aiming at lymphocytes infiltration for BC patients ante-lymphadenectomy, obtained AUCs up to 0.87 using a SIFT algorithm for classifying lymphocytes from non-lymphocytes. However, these studies involved smaller datasets and used different segmentation approaches and features with respect to this study. Moreover, they were published before the development of TILs-WG guidelines. Corredor et al. [12] performed a study aiming at detecting TILs on lung cancer H&E images using shape, texture, and color features. They also automatically detected nuclei with a watershed approach and obtained 89%, 83% and 86% in precision, recall and F1 score for TILs detection. Of note, these studies developed the classification approaches training SVM classifiers. In this study, for exploring the behavior of machine learning models that were suitable in case of training data much larger than the number of features, thus not exploring models that perform better with a low amount of training data and large features [48]. Moreover, differently from the above-mentioned studies, this work was focused on sTILs, according to the recommendations of TILs-WG [5]. Other studies aimed at developing DL-based computational TIL assessment methods broadly following some or all steps of visual TILs-WG guidelines [21,22,25]. Lu et al. [13] developed a U-Net model for lymphocyte detection showing 0.9536, 0.901, 0.9266 of recall, F1-score and precision, and strong association with immune response of outcomes. Le et al. [14], in a study aiming at exploring the correlation between tils distribution and invasive tumor through CNNs, obtained AUCs up to 0.950 for TILs. Sun et al. [21] developed a deep learning-based tool for an automatic til score assessment and achieved a F1-score of 0.856 for nuclei (also including TILs) classification. Similar approaches were also developed for detecting TILs in other cancer types [11,[49], [50], [51]].

Although the promising results achieved by DL-based approaches, a hand-crafted pathomics approach was preferred rather than a fully automated DL one as this could give better control over the initial data analysis and subsequent ML model construction and a better understanding of the whole pipeline. In fact, although the majority of DL-based methods have strong predictive performance and are comparable to the traditional hand-crafted approaches, these methods have limited ability to explain the deep features with neither a set of diagnostic plans nor an insight into the results. In particular, it was considered appropriate and constructive to explore hand-crafted pathomic features since pathomic and in general digital pathology is a recently explored field and, given the additional information related to the underlying biological processes that pathomics could provide, this study may add value in this direction. In this regard, while it might be difficult to demonstrate a causal relationship between pathomic features and underlying biological processes, it is possible to hypothesize about their underlying connections. It is worth noting that among features that passed the feature selection step and thus constitute ML models, there are features associated with shape (e.g. circularity, FSD) and intensity (mean, median absolute deviation and energy), and this could be related to the well-known properties of TILs of having a regular shape, clearer margins and higher peak intensity than other cells. Moreover, the contribution of features associated with gradient (mean, kurtosis) and Haralick features could be attributable to the more homogeneous enhancement and different textural distribution pattern of TILs than other cells [13]. Of note, Haralick features from nuclear were shown to be prognostic among different cancer types [52]. Despite some promising results emerging in this preliminary study, there are still many limitations which have to be acknowledged. The first concerns the unbalanced nature of the dataset, which has been tried to account for by applying SMOTE and downsampling techniques that have demonstrated their value in the setting of medical imaging ML analysis [44]. Moreover, although the dataset was very large given the high number of nuclei used for training and test models, the number of corresponding WSIs is from a limited number of patients. On a positive note, the study has the advantage of involving patients from different institutions.

Another issue concerns the prerequisite of pixel level annotation for individual nuclei segmentation within tumor-associated stroma, thus implying that any segmentation errors can have a direct impact on the final performance. Although the choice of an automatic and well-established approach guarantees robustness and reproducibility, the lack of a segmentation ground truth does not allow to quantitatively evaluate and control the segmentation error (e.g. through Dice index or F1-score [53,54]) and also to test more advanced segmentation methods (e.g. training neural networks), also by making comparisons (as done in previous studies) with other existing nuclear segmentation algorithms implemented in different software/studies [[55], [56], [57]].

It must be emphasized, however, that the problem of segmentation is inherent in hand-crafted approaches that aim to extract quantitative descriptors from regions of interest (e.g. radiomics, pathomics), and embraces all the advantages and disadvantages associated with the different segmentation techniques (manual, semi-automatic, automatic) [58].

It is worth noting that the implemented segmentation has the advantage of being fully automatic, and so not prone to inter- and intra-observer variability, replicable and not time-consuming for the pathologist [59].

As previously highlighted, a limited number of features was explored in this study to be able to interpret them directly and give a physical meaning as reliable as possible. An essential criterion for having examined these features is that they are easy to interpret, as opposed to features obtained from neural networks [20,22]. However, this framework could be used in future studies evaluating an expanded suite of pathomic measurements, as well as extended to characterizing TILs in other cancer types. For example, it might have been interesting to explore nuclear architectural information and local cell cluster graph-based measurements that have been reported to be of prognostic value in non-small cell lung cancer [60]. However, this is related to another issue concerning the missing information on both diagnostic and prognostic outcomes associated with patients (e.g., it is only known that the patients had HER2+ or TNBC immunophenotype, but the distribution was not specified; moreover, no prognostic outcomes were provided), which prevented us from performing diagnostic/prognostic predictions using the TIL assessment obtained using the developed classification method. Moreover, also based on previous studies tried to assess TILs using immunohistochemistry (IHC) images [[61], [62], [63]], it could be interesting to investigate pathomic features from IHC to add value to the developed approach. While TIL scoring in conventional H&E sections has the advantage that no additional stains are required, lymphocyte subtyping into cytotoxic, helper, regulatory, and other T cells not possible in H&E may become clinically relevant in the future. Therefore, in addition to TIL detection through morphological (H&E) features, subtyping of TILs through IHC could be relevant [9]. Another critical limitation affecting the study concerns the well-known lack of shared reference standards concerning data storage, the missing agreement on analysis procedures, and the feature reliability and reproducibility limitations affecting pathomics [64,65]. In particular, the existing lack of standardization in image acquisition, preprocessing, segmentation methods, and pathomics analysis tools, could lead to discrepancies in feature measurements that are not due to underlying biological variations [20,66]. However, all steps of pathomic workflow performed in this study were reported in detail since it is essential to develop this emerging field in terms of clinical translation and to improve the reproducibility of study outcomes [67]. It is worth noting that the proposed approach has followed a fragment of the TIL-WG guideline, by leveraging on annotations of tumor-associated stroma in which sTILs were annotated. In particular, having worked on the tumor-associated stroma annotations masked some intrinsic pitfalls associated with the identification of stromal regions (e.g. the exclusion of areas of necrosis, ductal and lobular carcinoma in situ (DCIS/LCIS), and normal breast tissue) [7]. Studies trying to address all the concepts of stromal and intratumoral TILs and accounting for confounding morphologies specific to different tumor sites, subtypes, and histologic patterns as envisioned by the TIL-WG are required [22]. In addition, it should also be highlighted that a general limitation of an automated approach for TIL evaluation concern whether its performance should be measured i) as the concordance between manual and automated sTIL score (namely the agreement between automatic TIL score and TIL score visually evaluated by pathologist or ii) according to the clinical outcome of the patient (e.g., the ability to predict survival or response to treatment, or a mix of both [14,21]. Although findings of this study require careful interpretation due to the limitations mentioned above, the proposed hand-crafted pathomic approach revealed interesting results that may represent a starting point for future research aiming at developing a quantitative, standardized, interpretable, automatic and as simple as possible method for TIL assessment. In the future, the generalizability of the proposed method should be validated on more datasets with larger WSI, incorporating it into a more complex pipeline that includes all the steps of the TIL-WG guidelines, also exploring the impact of different intermediate processing steps (e.g. nuclear segmentation, stain normalization) on the TILs classification performance.

## 5 Conclusions

The proposed pathomic approach accurately classified sTILs on H&E images of HER2+ and TNBC. This study brought to attention several types of quantitative features that are potentially easier to interpret and linked to underlying biological processes that can assist pathologists in improving diagnosis by increasing their understanding of the pathology. Compared with the state-of-the-art DL approaches, the proposed method is therefore an important step towards quantitative, reproducible, interpretable, and rater-independent TILs quantification analysis in BC histopathology and is also easily applicable to new datasets and domains.

## Declarations

### Author contribution statement

Valentina Brancato; Mario Verdicchio: Conceived and designed the experiments; Performed the experiments; Analyzed and interpreted the data; Contributed reagents, materials, analysis tools or data; Wrote the paper.

Carlo Cavaliere: Contributed reagents, materials, analysis tools or data; Analyzed and interpreted the data; Wrote the paper.

Francesco Isgrò; Marco Salvatore: Analyzed and interpreted the data; Wrote the paper.

Marco Aiello: Conceived and designed the experiments; Analyzed and interpreted the data; Wrote the paper.

## Funding statement

This work was partially funded by the Italian Ministry of Health (“Ricerca Corrente” project) and “5 × mille” funding for IRCCS SYNLAB SDN [Grant number: 2779020].

## Data availability statement

Data will be made available on request.

## Declaration of interest's statement

The authors declare no competing interests.
